# Early improvement of executive test performance during antidepressant treatment predicts treatment outcome in patients with Major Depressive Disorder

**DOI:** 10.1371/journal.pone.0194574

**Published:** 2018-04-18

**Authors:** Stefanie Wagner, Isabella Helmreich, Daniel Wollschläger, Konstantin Meyer, Sabine Kaaden, Julia Reiff, Sibylle C. Roll, Dieter Braus, Oliver Tüscher, Florian Müller-Dahlhaus, André Tadić, Klaus Lieb

**Affiliations:** 1 Department of Psychiatry and Psychotherapy, University Medical Centre, Mainz, Germany; 2 Institute of Medical Biostatistics, Epidemiology and Informatics (IMBEI), University Medical Centre, Mainz, Germany; 3 Sana Klinikum Offenbach, Offenbach, Germany; 4 Department of Psychiatry and Psychotherapy, HELIOS Dr. Horst-Schmidt-Kliniken, Wiesbaden, Germany; 5 Department of Psychiatry and Psychotherapy, Vitos Rheingau, Eltville, Germany; Brown University, UNITED STATES

## Abstract

Executive dysfunctions frequently occur in patients with Major Depressive Disorder and have been shown to improve during effective antidepressant treatment. However, the time course of improvement and its relationship to treatment outcome is unknown. The aim of the study was to assess the test performance and clinical outcome by repetitive assessments of executive test procedures during antidepressant treatment. Executive test performance was assessed in 209 –patients with Major Depressive Disorder (mean age 39.3 ± 11.4 years) and 84 healthy controls five times in biweekly intervals from baseline to week 8. Patients were treated by a defined treatment algorithm within the early medication change study (EMC trial; ClinicalTrials.gov NCT00974155), controls did not receive any intervention. Cognitive domains were processing speed, cognitive flexibility, phonemic and semantic verbal fluency. Intelligence was assessed at baseline. Depression severity was tested once a week by the Hamilton Depression Rating Scale (HAMD_17_). 130 patients (62%) showed executive dysfunctions in at least one of four tests at baseline. Linear mixed regression models revealed that the course of depression severity was associated to the course of cognitive flexibility (p = 0.004) and semantic verbal fluency (p = 0.020). Cognitive flexibility and semantic verbal fluency may be candidates easily to apply for therapy response prediction in clinical routine, which should be tested in further prospective studies.

Trial registration: ClinicalTrials.gov NCT00974155

EudraCT: 2008-008280-96

## Introduction

Major depressive disorder (MDD) is often accompanied by executive dysfunctions [1, 2]. In a meta-analysis on executive dysfunctions in unipolar, non-psychotic MDD patients we found that patients performed worse than healthy controls in all test procedures with the largest differences between patients and controls in inhibition capacity (effect size [ES]: 1.18), cognitive flexibility (ES: 1.11) and semantic verbal fluency (ES: 0.92) [3]. Imaging studies suggest that MDD associated executive deficits are the result of a frontal hypometabolism which can be normalised by antidepressants [4,5]. In line with that, inhibition capacity, verbal fluency and cognitive flexibility have been shown to improve during antidepressant therapy [3, 6]. However, a current meta-analysis on cognitive effects of antidepressants in depressed patients found no post-treatment differences in TMT B performance between antidepressant and placebo (SMD: 0.12, 95%-CI: -0.03–0.28) [7]. Previous studies investigating the effect of antidepressants on executive functions examined the test performance either in euthymic MDD patients compared to healthy controls [7] or before and after treatment [8–12]. In most of these studies, the time span between the two assessments was at least two months. The few studies assessing the test performance after three to four weeks reported a slight improvement of test performance, but the sample size was small and they used the same test version at admission and discharge [13, 14], increasing the risk of learning effects [15]. Thus, nothing is known about the detailed time course of executive test performance during antidepressant treatment.

In this study we describe the detailed course of executive functions and psychomotor speed during antidepressant treatment by repetitive measures of executive test performance and relating it to depressive symptomatology and treatment outcome. Additionally, test performance was assessed in 84 age- and sex-matched healthy controls in the same biweekly intervals.

## Materials and methods

This study was conducted between 2009 and 2013 in 226 MDD patients who have participated in the “Randomised clinical trial comparing an early medication change (EMC) strategy with treatment as usual (TAU) in patients with Major Depressive Disorders (MDD)—The EMC Trial” (ClinicalTrials.gov number: NCT00974155; EudraCT: 2008-008280-96). Details of the study protocol [16–18] and results of the EMC trial [19] have been described previously. In brief, the EMC Trial was a multi-center, randomized, controlled clinical trial investigating whether non-improver after 14 days of treatment with escitalopram are more likely to attain remission on treatment day 56 with an early medication change (immediate change to venlafaxine followed by an augmentation with lithium after non-response at day 28) compared to a treatment according to current guidelines (continuing escitalopram for 2 weeks followed by venlafaxine).

All participants gave their written informed consent to participate in the study after a complete and extensive description. All study components were approved by the local ethical committee of the Landesärztekammer Rheinland-Pfalz (study code n°: 837.166.09 (6671)) and are compliant with the Code of Ethics of the World Medical Association (Declaration of Helsinki) in its current version.

The neuropsychological investigations were conducted in two of the eight study centres. The healthy volunteers were recruited by posters, which were hanging up in the University Medical Centre in Mainz.

Key inclusion criteria of the EMC trial were: 1) Major Depressive Disorder (MDD), first episode or recurrent, according to DSM-IV [20], 2) HAMD_17_ score of ≥18 at screening; 3) age 18–65 years and ≤60 years at first depressive episode; key exclusion criteria: 1) Bipolar Disorder or psychotic depression; 2) benzodiazepines > 1.5 mg Lorazepam; 3) no native speaking German.

### Study procedures

The existence of mental or personality disorders was assessed by the German versions of the M.I.N.I. International Neuropsychiatric Interview (MINI) [21] and the Structured Clinical Interview for DSM-IV Axis II Personality Disorders (SCID-II) [22]. Physical disorders were assessed by the German version of the Cumulative Illness Rating Scale [23].

Depression severity was assessed by the German version of the Hamilton Depression Rating Scale (HAMD-17) [24] in weekly intervals. In each of the two study centres, three different blinded raters were involved in the assessment of the clinical characteristics and neuropsychological tests. All raters were trained in the use of the psychometric scales (MINI, SCID-II, HAMD) as well as the neuropsychological test procedures before the beginning of the study. Inter-rater reliablility was high between the different raters [25].

Test performance was assessed at baseline, day 14, 28, 42 and 56. The investigated cognitive functions were psychomotor speed (Trail Making Test [TMT] A) [26], cognitive flexibility (TMT B), semantic and phonemic verbal fluency (Regensburger Wortflüssigkeitstest [RWT]) [27]. The TMT and RWT were administered five times in biweekly intervals from baseline to week 8. In TMT A, subjects have to draw lines to connect 25 numbers in ascending order. In part B, patients have to draw lines alternating between numbers and letters. The measure of performance is the time a patient needs to connect all circles. Because the TMT originally existed only in 1 version, we developed and validated three alternate forms in a previous study [28]. The four alternate forms were randomly distributed to patients and visits 1 to 4, at day 56 the version administered at baseline was repeated. The difference between TMT A and B (B-A) was calculated as it was proofed to be an important index of cognitive flexibility [29, 30].

In phonemic verbal fluency tasks, participants have to generate words beginning with a specific letter; in the semantic fluency tasks, subjects are instructed to generate as many words (e.g. dog) as possible belonging to a specific semantic category (e.g. animals). The measure of performance is the number of correct words given in 2 minutes. The raw scores were transferred into age-corrected percentiles for each letter or category. The RWT consists of 5 alternate forms which were randomly distributed to patients and visits.

General intelligence was examined once at baseline using the Multiple Vocabulary Test (MWT-B) [31]. The raw scores of the MWT-B were transferred into IQ values (mean = 100, SD = 15).

### Sample size calculation

Pre-study sample size calculation was based on a two-sided Chi^*2*^-test for the question whether patients with an improvement of the test performance in the first two weeks of treatment (group 1) more often become remitters than patients without an improvement of the test performance (group 2). Basing on treatment response rates of early improver and non-improver, we assume a treatment response rate of 0.5 in group 1 and of 0.2 in group 2 resulting in an odds ratio of 0.250. Assuming an allocation ratio between group 1 and 2 of 0.54 (~1:1.85), the sample size calculation yields a sample size of 128 patients (83 in group 1, 45 in group 2) to reach a power of 90% with a significance level of of α = 0.05. Assuming a drop-out rate of 15%, the total sample size for the study was calculated with 148 patients.

Differences in the test performance between patients and controls were calculated by 2-sided t-tests for independent groups with a significance level of α = 0.05. Aiming a power of 90% and an effect size of 0.80, 50 healthy controls had to be investigated. Assuming a drop-out rate of 25% in healthy controls because of the extensive measures and the large number of visits by a lack of direct utility for the volunteers, 70 healthy controls had to be included in the study.

### Statistics

Data were analyzed using IBM SPSS Statistics 23.0. Statistical significance was set at p ≤ 0.050.

An impaired test performance was defined as a performance ≤ 1 standard deviation (SD) below the mean of patients´ age group (percentile ≤ 16) according to relevant norm values [26, 32, 33]. To identify the impact of potential covariates on test performance, we calculated correlation analysis between test scores at baseline and the covariates age, intelligence (Pearson correlation coefficient) and sex (Spearman´s rank correlation coefficient).

Differences between patients and controls were examined by repeated-measures ANCOVAs with the significant parameters from the correlation analysis as covariates (age, intelligence). Important results in this analysis are the main effect for time (BL, day 14, 28, 42 & 56) and for groups (patients versus controls) and the interaction effect time X groups. In this context, a significant Time X group interaction means that the effect of time depends on whether the subject was a depressed patient or a healthy control subject.

Linear mixed effects regression models were used to investigate how HAMD sum scores developed over the follow up period of 8 weeks depending on executive function measures while allowing for random individual intercepts. In the first model, HAMD sum scores for all 5 time points (BL, day 14, 28, 42, 56) were modeled using scores on the difference TMT B-A, phonemic and semantic verbal fluency for all 5 time points as time-varying covariates while adjusting for baseline age and IQ. The model also included a natural spline term with 3 degrees of freedom for day of follow up to allow for non-constant change in HAMD scores.

In the second model, change in HAMD sum scores relative to baseline were modeled using only baseline scores for the difference TMT B-A, phonemic and semantic verbal fluency, age, and IQ as static covariates as well as a linear term for day of follow up as a time-varying covariate. F-tests with Kenward-Roger corrected degrees of freedom were used to assess statistical significance of covariates [34]. Marginal and conditional R^2 was calculated to assess overall model fit [35]. As a measure for the correlation between two HAMD observations from the same individual, the intra-class correlation coefficient (ICC) was calculated as the ratio of random intercept variance to total variance. Linear mixed effects models were fitted in the statistical environment R [36] using packages lme4 [37] and pbkrtest [38].

In exploratory analyses we calculated the number of patients with or without executive dysfunctions at baseline. In a second step, we analyzed the number of patients with executive dysfunctions at baseline, but either a normalization or a persistence of these deficits until day 56. Executive dysfunctions were defined as a performance ≤ 1 standard deviation (SD) below the mean of the age group (percentile ≤ 16) according to relevant norm values [27, 32–33]. A normalization was defined as a test performance of percentile >16 at day 56. Differences in mean test performance between patients receiving escitaloptam, venlafaxine or venlafaxine and lithium were calculated by one-way ANOVA.

## Results

**Fig 1:**
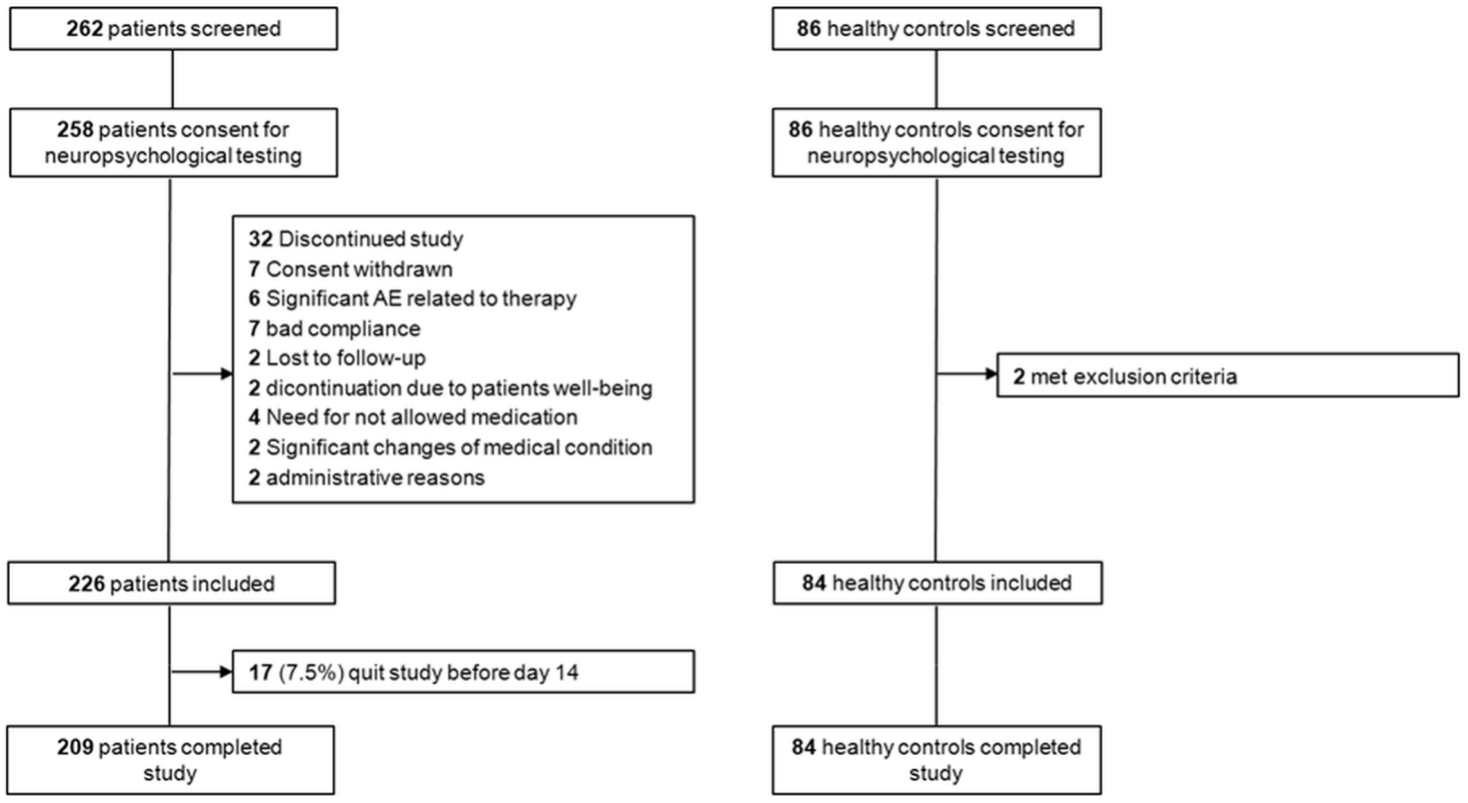
CONSORT flow chart. AE: adverse events.

226 patients were eligible for analysis, of which 17 (7.5%) did not finish the study. Thus, complete data of 209 patients were available. Additionally, 84 healthy control subjects were analyzed (for detail see Fig 1). Demographic and clinical data of patients and controls are given in Table 1. Patients had significantly lower IQ-values than controls (p≤0.001), but were not significantly different to controls with respect to sex and age (for detail see Table 1).

**Table 1:** Demographic and clinical features of patients and controls.

features		MDD patients (N = 209)	Controls (N = 84)	p value*
Sex [n(%)]	*Male* *Female*	*105 (50.2)* *104 (49.2)*	*35 (41.7)* *49 (58.3)*	0.198
Education	No Lower secondary education High-school diploma Technical college Academic high school	*1 (0.5)* *45 (21.5)* *60 (28.7)* *23 (11.0)* *78 (37.3)*	*1 (1.2)* *6 (7.1)* *18 (21.4)* *9 (10.7)* *50 (59.6)*	0.009
*Vocational* education	No Apprenticeship Foreman Scholastics *Other*	*23 (11.0)* *101 (48.3)* *4 (1.9)* *79 (37.8)* *2 (1.0)*	*9 (10.7)* *28 (33.3)* *1 (1.2)* *45 (53.6)* *1 (1.2)*	0.001
Median Age (range) [years]		40.0 (18–64)	31.0 (20–63)	0.117
		**Mean ± SD**	**Mean ± SD**	
Age [years]		39.3 ± 11.4	36.4 ± 12.8	0.060
Intelligence		104.0 ± 14.2	112.1 ± 11.7	<0.001
HAMD-17 sum score at BL		23.0 ± 5.1	0.7 ± 1.1	<0.001
Course of depression [n(%)]	First episode Recurrent MDD	*85 (40.7)* *124 (59.3)*		
Remitter at day 56 (HAMD < 7)	Remitter Non-remitter	*87 (44.4)* *111 (55.6)*		
Previous medication	Yes No	*99 (47.4)* *110 (52.6)*		
		**Mean ± SD**		
Age at onset [years]		32.1 ± 12.2		
Number of previous episodes		3.6 ± 3.8		
Duration of current episode [weeks]		29.7 ± 50.9		
CIRS sum score		*3.1 ± 2.3*		

* *independent t-test* or Chi^*2*^-test; MDD: Major Depressive Disorder; HAMD: Hamilton Depression Rating Scale; BL: baseline; EP: endpoint, SD: standard deviation; CIRS: Cumulative Illness Rating Scale

The correlation analyses showed that in both groups at baseline, older participants were slower in TMT than younger ones (**A**: r = 0.37; p<0.001; **B**: r = 0.42; p<0.001). In patients, age was also associated to semantic verbal fluency performance (r = 0.17; p = 0.017). Intelligence positively correlated with phonemic (r = 0.39; p<0.001) and semantic verbal fluency performance (r = 0.25; p = 0.003) in patients and controls. Sex was not associated with test performance (p≥0.12).

### Course of executive test performance of patients and controls

**Fig 2:**
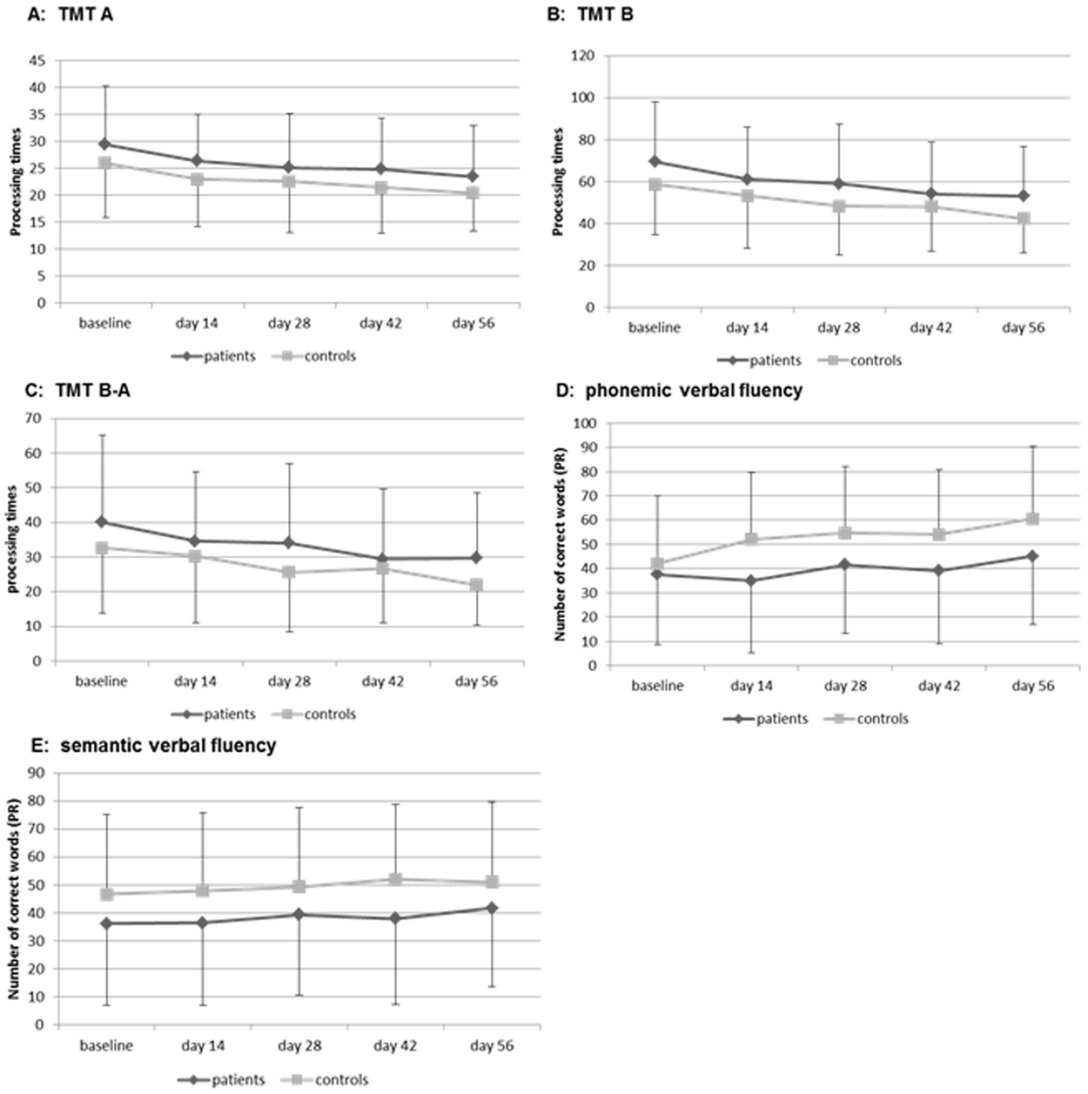
Course of executive test performance in patients (N = 209) and controls (N = 84). TMT: Trail Making Test; PR: percentiles; * p < 0.050; ** p < 0.010; ***p < 0.001; repeated measures ANCOVA.

The repeated measures ANCOVA showed no differences in the course of the TMT performance between patients and controls (TMT A: main effect group: p = 0.370; main effect time: p = 0.005; TMT B: main effect group: p = 0.207; main effect time: p = 0.242; for detail see Fig 2A and 2B). There were no interaction effects between time (TMT performance from BL to day 56) and groups [patients vs. controls] (TMT A: p = 0.755; TMT B: p = 0.425).

The phonemic (main effect group: p = 0.773, main effect time: p = 0.337) and semantic verbal fluency performance (main effect group: p = 0.962; main effect time: p = 0.598) also did not differ between patients and controls (for detail see Fig 2C and 2D and Table B in S1 File), nor was there an interaction effect between time (verbal fluency performance from BL to day 56) and groups [patients vs. controls] (phonemic: p = 0.498; semantic: p = 702). Effect sizes for the comparison of the test performance between patients and controls were low to moderate (Cohen´s d: 0.26–0.54; for detail see Table C in S1 File, for individual data see S2 File).

### Number of patients with and without impairment in executive test performance and normalization of executive test performance in patients with deficits at baseline

**Fig 3:**
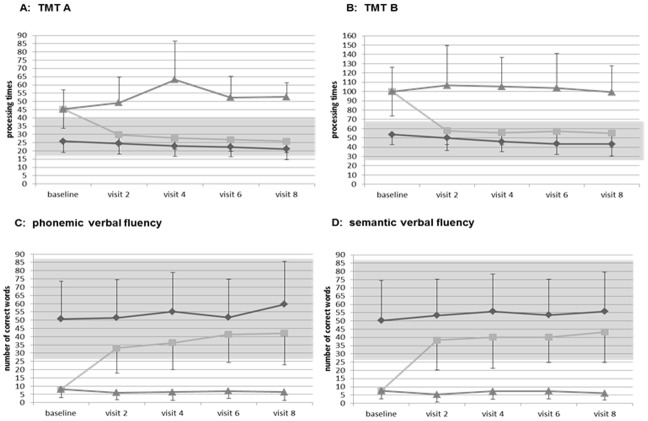
TMT test performance in patients with no cognitive impairment (“not impaired”) and in patients with cognitive impairment at baseline which normalized (“normalized”) or did not normalize (“impaired”) at endpoint. TMT: Trail Making Test.

130 patients (62%) showed deficits in at least one of the four tests at study initiation, 84 (40%) patients had impairment in one of the TMT subtests, 101 (48%) in at least one verbal fluency task. From the 130 patients with executive dysfunction at baseline, 85 (65%) experienced a normalization of the impaired test performance (normalization in TMT A: 28 (85%), B: 44 (74%), B-A: 45 (69%), phonemic fluency: 38 (70%), semantic fluency: 30 (63%)), while a subset of patients (45, 35%) showed persisting executive deficits in at least one test until day 56. A normalization was defined as a test performance of percentile >16 at day 56. Patients with a normalization achieved a test performance within the normal range (mean ± 1 SD or above) of their age group (18–29, 30–41, 42–53 & 54–65 years) according to relevant norm values from day 14 onwards. Patients with persisting deficits performed worse than the other patients in all test procedures (for illustration see Fig 3A and 3B for TMT & Fig 2C and 2D for verbal fluency).

### Course of depression severity depending on test performance

The first model of the linear mixed effects regression showed that the HAMD scores for all 5 time points were significantly associated with the difference TMT B-A, semantic verbal fluency performance and intelligence (for detail see Table 2), but not with phonemic verbal fluency or age. Patients with a better performance in these subtests had lower HAMD sum scores during study. Additionally, patients with higher IQ values had higher depression severity scores. Intra-class correlation coefficient for this model was ICC = 0.491, marginal R^2 = 0.396 and conditional R^2 = 0.693. In the second model we found that the change in HAMD sum scores relative to baseline was not significantly associated with any of the baseline variables (ICC = 0.697, marginal R^2 = 0.082, conditional R^2 = 0.722; for detail see Table 2).

**Table 2:** Development of HAMD sum scores over the follow up period of 8 weeks depending on the course of executive function measures as well as age and IQ.

Covariate	estimate (β)	standard error	*df effect*^*1*^	*df error*^*2*^	t-value	p-value*
*Course of test performance*						
Intelligence	0.086	0.028	*1.00*	*219.83*	3.08	0.002
Age	-0.027	0.035	*1.00*	*207.87*	-0.76	0.450
Trail Making Test (TMT B-A)	0.029	0.010	*1.00*	*929.07*	2.87	0.004
Phonemic verbal fluency	-0.001	0.009	*1.00*	*923.76*	-0.13	0.900
Semantic verbal fluency	-0.019	0.008	*1.00*	*911.02*	-2.34	0.020
*Performance at baseline*						
Time	-0.118	0.009	-12.75	*1.00*	*189.58*	<0.001
Intelligence	0.036	0.038	0.94	*1.00*	*189.10*	0.350
Age	-0.054	0.046	-1.17	*1.00*	*563.00*	0.240
Trail Making Test (TMT B-A)	0.018	0.021	0.88	*1.00*	*188.08*	0.380
Phonemic verbal fluency	-0.037	0.021	-1.77	*1.00*	*188.34*	0.078
Semantic verbal fluency	-0.013	0.019	0.68	*1.00*	*189.38*	0.500

* F-tests with Kenward-Roger corrected degrees of freedom (Linear mixed effects regression models); *df*: *degree of freedom; Non-integer df due to Kenward-Roger correction for*^*1*^*effect and*^*2*^*error*

### Effect of medication on test performance

Patients receiving Venlafaxine and Lithium had lower processing times in TMT A at day 56 than patients treated with Escitalopram or Venlafaxine (p = 0.005, for detail see Table 3). In part B of the TMT as well as in verbal fluency, patients receiving different antidepressants did not differ in their mean test performance (TMT B: p = 0.063; phonemic: p = 0.156; semantic: p = 0.350). Patients receiving a concurrent medication with benzodiazepines did not differ in their test performance from patients who did not take any benzodiazepines (TMT A: p = 0.148; TMT B: p = 0.933; phonemic: p = 0.486; semantic: p = 0.675).

**Table 3:** Mean test performance and depression severity at day 56 separated for study medication.

	Escitalopram (N = 73)	Venlafaxine (N = 78)	Lithium (N = 17)	p-value*
	Mean ± SD	Mean ± SD	Mean ± SD	
Trail Making Test (TMT A)^1^	22.0 ± 6.1	22.8 ± 9.6	29.4 ± 12.8	0.007
Trail Making Test (TMT B)	50.3 ± 16.7	51.6 ± 26.7	64.8 ± 27.9	0.063
Phonemic verbal fluency	46.3 ± 31.3	48.7 ± 28.9	33.1 ± 31.0	0.156
Semantic verbal fluency	42.6 ± 28.8	42.8 ± 29.3	32.0 ± 25.3	0.350
HAMD_17_ sum score	5.0 ± 4.4	11.1 ± 6.6	15.8 ± 7.7	0.000

^1^ According to the treatment algorithm of the EMC *trial*, all patients received Escitalopram for the first 2 weeks. In case of non-improvement (day 14) or non-response (day 28), they were switched to Venlafaxine and further on augmented with Lithium in case of non-improvement to Venlafaxine (day 28 or 42). Thus, the first time point to compare the test performance between the 3 antidepressants is day 56;

* one-way ANOVA; SD: standard deviation; HAMD: Hamilton Depression rating Scale

## Discussion

We applied for the first time repetitive measures of executive test performance in MDD patients and healthy controls during a period of eight weeks. Due to the biweekly assessment of test performance and depression severity, the study design enabled us to determine the detailed time course of executive test performance during treatment and to analyse the association between executive test performance and depressive symptomatology.

We found that in the total group patients did not perform worse than controls in all executive tests. However, 130 (62%) patients showed impaired test performance in at least one of the four tests at treatment initiation. This percentage of impaired patients [9, 10, 12] as well as effect sizes differences in test performance between patients and controls are in line with previous studies [1–3]. Estimations of the degree of cognitive impairments in depression indicate a statistically moderate magnitude of cognitive impairment. In a meta-analysis by Christensen et al. [39], a deficit in cognitive functions of, on average, 0.63 standard deviations below that of healthy controls has been reported in patients with depression, while the most impaired cognitive function (flexibility) was more than one standard deviation lower than the healthy controls’ performance [13]. Furthermore, studies report that 21% of patients with unipolar depression demonstrate more severe cognitive impairment (defined as test performance at least two standard deviations below normative values) in at least two cognitive domains which is only found in 4% of healthy controls [40].

The linear mixed regression effects model suggests that the course of the HAMD sum scores over the follow up period of 8 weeks depends on the course of cognitive flexibility (TMT B-A) and semantic verbal fluency performance as well as on intelligence at baseline. If the change of cognitive flexibility and semantic verbal fluency could be confirmed as a predictor for treatment outcome in MDD in future investigations, they may be candidates easily to apply in the prediction of therapy response. The linear mixed model further revealed that the change of the HAMD sum scores during the study could not be predicted by the baseline executive test performance. This result suggests that the development or increase of the executive test performance during treatment seems to be an important variable for the prediction of later treatment outcome. The advantage of our study was the repeated assessment of the test performance which revealed an association between the course of depression severity and the performance in cognitive flexibility and semantic verbal fluency. Since the largest change in test performance occurred in the first 2 weeks, cognitive flexibility and semantic verbal fluency may be possible predictors for later treatment outcome. This appears to be interesting in the light of studies indicating that antidepressants start to exert their efficacy as early as during the first two weeks of treatment [41] and that an effective antidepressant treatment leading to an early improvement of depressive symptomatology successfully triggers molecular and cellular downstream effects enabling normalization from the disorder [42, 43]. Whether the early improvement of executive functions is triggered by the same mechanisms as the early improvement of depressive symptoms is unclear. However, our data show that the normalization of executive dysfunctions went mainly in parallel with the improvement in depression scores and recent imaging data in depressed patients have shown that similar networks are active in depression and cognition [4, 5]. Both findings support the hypothesis of similar underlying mechanisms leading to early improvement of depressive symptomatology and executive functions.

Looking at the test performance at the end of therapy, we found that 65% of patients with executive deficits at baseline showed a normalization of their test performance until day 56, while 35% of patients still showed abnormal test performance in at least one test, with a higher percentage of patients with persisting deficits in verbal fluency (21%) than in processing speed and cognitive flexibility (11%). The number of patients with executive dysfunctions at endpoint is comparable to previous studies, which also found a higher percentage of patients with deficits in verbal fluency tasks than in processing speed and cognitive flexibility [9, 10]. A previous study of Shilyansky and colleagues (2016) [44] found an improvement in executive functions and cognitive flexibility during an antidepressant treatment, specifically in patients with a remission of the depressive symptomatology. The examination of demographic characteristics of patients with persisting deficits showed that older patients were less likely to experience a normalization of their executive deficits than younger patients, which is in line with studies showing that cognitive dysfunctions are a core symptom of older depressed patients possibly because of a loss of cognitive reserve [45]. As in previous studies, patients with higher premorbid intelligence scores seem to have lower deficits in verbal fluency tasks [2, 46]. Since, depressed patients with neuropsychological deficits tend to show less compliance with antidepressant treatment, show an increased risk for suicide and a reduced level of psychosocial and occupational functioning, the identification of this subgroup and the development of effective treatment strategies for depression as well as neuropsychological deficits are needed [40].

### Effect of medication on test performance

The results showed that patients receiving a combination of venlafaxine and lithium performed significantly worse in part A of the TMT than patients receiving escitaopram or venlafaxine alone. This is in line with previous studies suggesting impaired psychomotor speed in patients treated with lithium [47]. Since patients treated with venlafaxine plus lithium had significantly higher depression severity scores, it remains unclear if the worse TMT A performance is the result of the medication or the more pronounced depressive symptomatology. From our point of view, the relation between the processing speed performance and medication did not affect the interpretation of our study results, because patients received the combination of venlafaxine and lithium only in the last 2 weeks of treatment and the number of these patients was very small (N = 17).

### Strengths and limitations

Strengths of our study are the repetitive administration of the test performance in parallel to the antidepressant treatment, the use of alternate test forms to reduce practice effects and the comparison to a large control group of matched healthy adults in order to separate treatment from practice effects. This comparison yielded low to moderate improvement of the test performance (Cohen´s d: 0.210 to 0.525) in healthy volunteers, but large improvement in all test procedures in patients (d = 0.980 to 1.163). This suggests that the observed improvement in patients is the result of treatment rather than practice effects.

A limitation of the study was that the mean intelligence (IQ = 112 pts.) of the control sample but not of the patients (IQ = 104 pts.) was in the upper range of the average of the general population. This might have led to greater differences between patients and controls as they would have been observed if the groups had similar IQ values. However, ANCOVAs were used to adjust the test performance from possible distortive intelligence effects, and the differences between patients and controls were comparable to previous studies [2, 3, 33]. Furthermore, the investigation of the subgroups might have created a regression to the mean, overestimating the improvement of test performance in the subgroups. Another limitation is that we only include four executive domains in our study. As a consequence our results are not generalizable to other executive functions. However, we only selected test procedures, which could be easily implemented in clinical routine and, additionally, are available in different alternate forms in case of repetitive assessments of the test procedures during an antidepressant treatment. The selected test procedures can be easily implemented in clinical routine, because it needs only a short training in the admission of the tests and their competition takes only 2 to 5 minutes. Cognitive flexibility is a traditional, specific task to assess executive functions. Verbal fluency tasks on the other hand are more complex neuropsychological tests (“compound tasks”) taping a wide variety of cognitive processes, including not only multiple aspects of executive functions (e.g., shifting between subcategories, working memory for what items have already been named, inhibitory processes), but also non-executive abilities (e.g., semantic processing). However, verbal fluency tasks are amongst the most widely used measures to assess executive functioning. This complexity of the task make it difficult to interpret, because it remains unknown if the impairments arise from deficits in shifting, working memory, or non-executive function aspects of the tasks, or some mixture of these factors. These concerns can in future studies be addressed by additionally using tasks designed to more specifically place demands on individual aspects of executive functions [48].

### Conclusion

This is the first study investigating the detailed time course of executive test performance parallel to an antidepressant therapy and a group of healthy controls. We were able to show that the change of depression severity depends on the increase of cognitive flexibility and semantic verbal fluency performance during the study. Cognitive flexibility and semantic verbal fluency may be candidates easily to apply in the prediction of therapy response which should be tested in future studies.

## Supporting information

S1 File Supporting information. (DOC)

S2 File Individual data for patients and controls. (XLSX)
