# Supplementary material for: Early improvement of executive test performance during antidepressant treatment predicts treatment outcome in patients with Major Depressive Disorder
Source: PLoS One. 2018 Apr 18;13(4):e0194574. doi: 10.1371/journal.pone.0194574 (PMC5905973; doi:10.1371/journal.pone.0194574)
Supplement: S1 File — (DOC) [file pone.0194574.s001.doc]

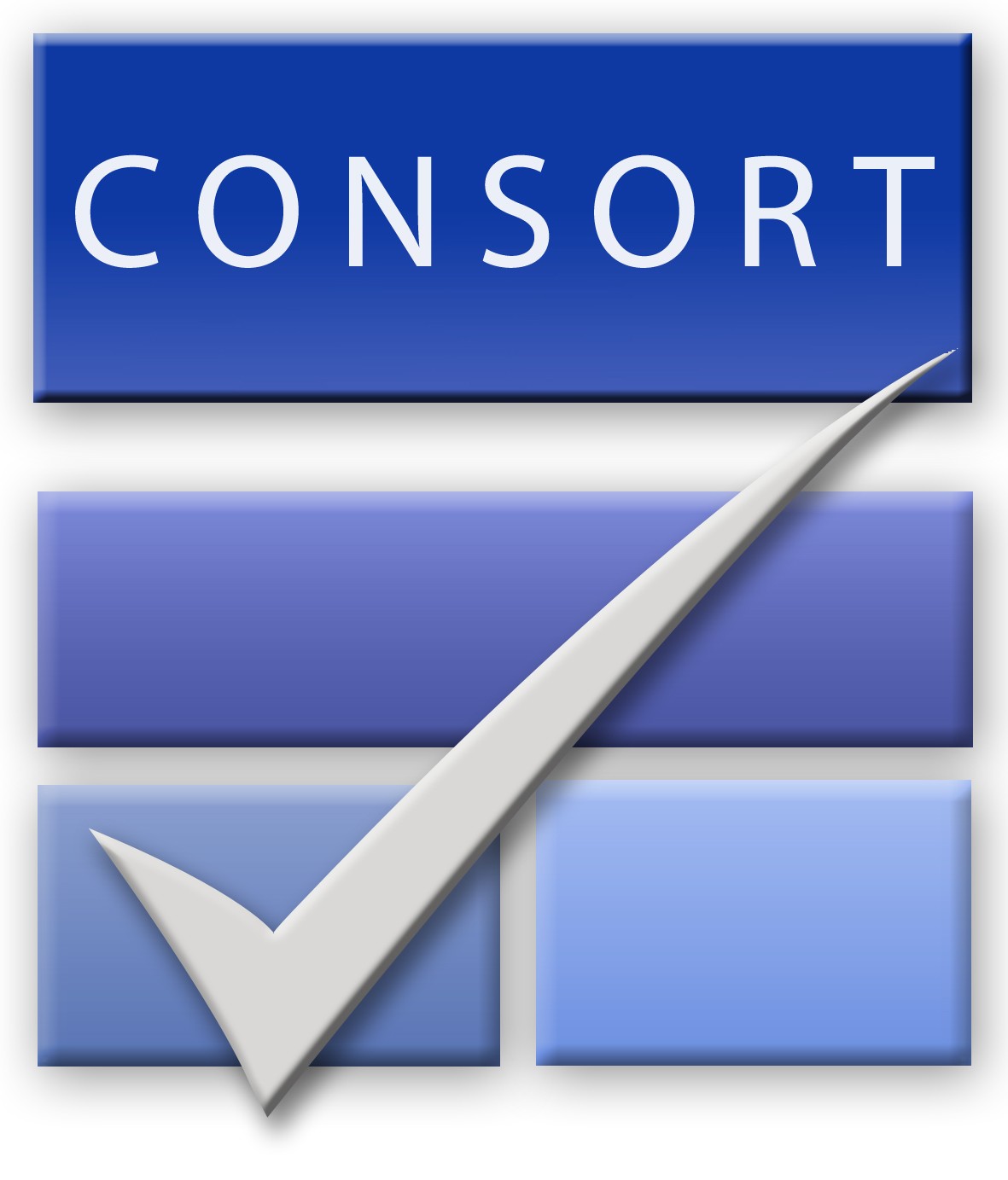
Supplementary table A: CONSORT 2010 checklist of information to include when reporting a randomised trial*

| Section/Topic | Item No | Checklist item | Reported on page No |
| --- | --- | --- | --- |
| Title and abstract | | | |
|  | 1a | Identification as a randomized trial in the title | Title page |
|  | 1b | Structured summary of trial design, methods, results, and conclusions (for specific guidance see CONSORT for abstracts) | 1 |
| Introduction | | | |
| Background and objectives | 2a | Scientific background and explanation of rationale | 2 |
|  | 2b | Specific objectives or hypotheses | 2 |
| Methods | | | |
| Trial design | 3a | Description of trial design (such as parallel, factorial) including allocation ratio | 3 |
|  | 3b | Important changes to methods after trial commencement (such as eligibility criteria), with reasons | n.a |
| Participants | 4a | Eligibility criteria for participants | 3 |
|  | 4b | Settings and locations where the data were collected | 3 |
| Interventions | 5 | The interventions for each group with sufficient details to allow replication, including how and when they were actually administered | 3-4 |
| Outcomes | 6a | Completely defined pre-specified primary and secondary outcome measures, including how and when they were assessed | 4-5 |
|  | 6b | Any changes to trial outcomes after the trial commenced, with reasons | n.a. (see related manuscript file) |
| Sample size | 7a | How sample size was determined | On page 3, we refer to the trial protocol (ref. 27-29) |
|  | 7b | When applicable, explanation of any interim analyses and stopping guidelines | n.a. (see related manuscript file |
| Randomisation: |  |  |  |
| Sequence generation | 8a | Method used to generate the random allocation sequence | n.a. (see related manuscript file) |
|  | 8b | Type of randomisation; details of any restriction (such as blocking and block size) | n.a. (see related manuscript file) |
| Allocation concealment mechanism | 9 | Mechanism used to implement the random allocation sequence (such as sequentially numbered containers), describing any steps taken to conceal the sequence until interventions were assigned | n.a. (see related manuscript file) |
| Implementation | 10 | Who generated the random allocation sequence, who enrolled participants, and who assigned participants to interventions | n.a. (see related manuscript file) |
| Blinding | 11a | If done, who was blinded after assignment to interventions (for example, participants, care providers, those assessing outcomes) and how | 4 |
|  | 11b | If relevant, description of the similarity of interventions | n.a. |
| Statistical methods | 12a | Statistical methods used to compare groups for primary and secondary outcomes | 5 |
|  | 12b | Methods for additional analyses, such as subgroup analyses and adjusted analyses | 5 |
| Results | | | |
| Participant flow (a diagram is strongly recommended) | 13a | For each group, the numbers of participants who were randomly assigned, received intended treatment, and were analysed for the primary outcome | Suppl. Figure 1 |
|  | 13b | For each group, losses and exclusions after randomisation, together with reasons | Suppl. Figure 1 |
| Recruitment | 14a | Dates defining the periods of recruitment and follow-up | 3 |
|  | 14b | Why the trial ended or was stopped | n.a. |
| Baseline data | 15 | A table showing baseline demographic and clinical characteristics for each group | Table 1 |
| Numbers analysed | 16 | For each group, number of participants (denominator) included in each analysis and whether the analysis was by original assigned groups | Figure 1-3 |
| Outcomes and estimation | 17a | For each primary and secondary outcome, results for each group, and the estimated effect size and its precision (such as 95 confidence interval) | 6-10; figure 1-3 |
|  | 17b | For binary outcomes, presentation of both absolute and relative effect sizes is recommended | 6-10, figure 1-3 |
| Ancillary analyses | 18 | Results of any other analyses performed, including subgroup analyses and adjusted analyses, distinguishing pre-specified from exploratory | 6-10 |
| Harms | 19 | All important harms or unintended effects in each group (for specific guidance see CONSORT for harms) | n.a. |
| Discussion | | | |
| Limitations | 20 | Trial limitations, addressing sources of potential bias, imprecision, and, if relevant, multiplicity of analyses | 13 |
| Generalisability | 21 | Generalisability (external validity, applicability) of the trial findings | 11-14 |
| Interpretation | 22 | Interpretation consistent with results, balancing benefits and harms, and considering other relevant evidence | 11-14 |
| Other information | | |  |
| Registration | 23 | Registration number and name of trial registry | 1 |
| Protocol | 24 | Where the full trial protocol can be accessed, if available | Relevant parts of the clinical trial protocol have been published in Tadic et al 2010, Tadic, Wagner et al 2011 (ref. 27-29) |
| Funding | 25 | Sources of funding and other support (such as supply of drugs), role of funders | 15-16 |

| **Supplementary table B: Mean test performance of patients and controls (for individual data see S2 file)** | | | | | | | | | | | | | | | |  | | |  | | |  | | |  | |  | |  | |  |
| --- | --- | --- | --- | --- | --- | --- | --- | --- | --- | --- | --- | --- | --- | --- | --- | --- | --- | --- | --- | --- | --- | --- | --- | --- | --- | --- | --- | --- | --- | --- | --- |
|  |  | | |  | | |  | | |  | | |  | | |  | | |  | | |  | | |  | |  | |  | |  |
|  | **Trail Making Test A** | | | | | | | | | | | **Trail Making Test B** | | | | | | | | | | | **Trail Making Test B-A** | | | | | | | | |
|  | MDD patients  (N=209) | | | | | healthy controls (N=84) | | | | | | MDD patients  (N=209) | | | | | healthy controls (N=84) | | | | | | MDD patients  (N=209) | | | | | healthy controls (N=84) | | | |
|  | mean | | SD | | | mean | | | SD | | | mean | | SD | | | mean | | | SD | | | mean | | | SD | | mean | | SD | |
| baseline | 29.4 | | 10.9 | | | 25.9 | | | 10.0 | | | 69.4 | | 28.9 | | | 58.6 | | | 24.1 | | | 40.1 | | | 25.1 | | 32.7 | | 18.8 | |
| day 14 | 26.3 | | 8.7 | | | 22.9 | | | 8.8 | | | 61.1 | | 24.9 | | | 53.2 | | | 24.9 | | | 34.6 | | | 20.1 | | 30.3 | | 19.2 | |
| day 28 | 25.1 | | 9.9 | | | 22.5 | | | 9.4 | | | 59.0 | | 28.3 | | | 48.2 | | | 23.3 | | | 34.0 | | | 22.9 | | 25.7 | | 17.3 | |
| day 42 | 24.8 | | 9.5 | | | 21.4 | | | 8.4 | | | 54.2 | | 24.5 | | | 48.1 | | | 21.3 | | | 29.5 | | | 20.2 | | 26.8 | | 15.6 | |
| day 56 | 23.4 | | 9.5 | | | 20.4 | | | 7.1 | | | 53.1 | | 23.5 | | | 42.3 | | | 16.2 | | | 29.7 | | | 18.8 | | 21.9 | | 11.6 | |
|  |  | | |  | | |  | | |  |  | | | | |  | | |  | | |  | |  | | |  | |  | |  |
|  | **phonemic verbal fluency** | | | | | | | | | | **semantic verbal fluency** | | | | | | | | | | | | |  | | |  | |  | |  |
|  | MDD patients (N=209) | | | | healthy controls (N=84) | | | | | | MDD patients  (N=209) | | | | | | | healthy controls (N=84) | | | | | |  | | |  | |  | |  |
|  | mean | SD | | | mean | | | SD | | | mean | | | | SD | | | mean | | | SD | | |  | | |  | |  | |  |
| baseline | 37.5 | 27.9 | | | 42.0 | | | 28.9 | | | 36.2 | | | | 28.4 | | | 46.7 | | | 29.2 | | |  | | |  | |  | |  |
| day 14 | 34.9 | 27.6 | | | 52.0 | | | 29.6 | | | 36.5 | | | | 27.9 | | | 47.9 | | | 29.5 | | |  | | |  | |  | |  |
| day 28 | 41.5 | 27.5 | | | 54.6 | | | 28.3 | | | 39.4 | | | | 28.4 | | | 49.4 | | | 28.9 | | |  | | |  | |  | |  |
| day 42 | 39.1 | 26.7 | | | 54.0 | | | 30.1 | | | 37.9 | | | | 26.7 | | | 51.9 | | | 30.6 | | |  | | |  | |  | |  |
| day 56 | 45.1 | 29.8 | | | 60.5 | | | 28.0 | | | 41.7 | | | | 28.7 | | | 50.9 | | | 28.1 | | |  | | |  | |  | |  |

**Legend to supplementary table B**: N= number; SD = standard deviation

**Supplementary table C: Effect sizes (Cohen´s d) for the comparison of patients and controls**

|  | **baseline** | **Visit 2** | **Visit 4** | **Visit 6** | **Visit 8** |
| --- | --- | --- | --- | --- | --- |
| MDD patients versus controls | | | | | |
|  | Cohen´s d (95%-CI) | Cohen´s d (95%-CI) | Cohen´s d (95%-CI) | Cohen´s d (95%-CI) | Cohen´s d (95%-CI) |
| Trail Making Test A | 0.33  (0.07 – 0.59) | 0.39  (0.13 – 0.65) | 0.26  (0.02 – 0.52) | 0.38  (0.12 – 0.64) | 0.35  (0.09 – 0.61) |
| Trail Making Test B | 0.39  (0.14 – 0.66) | 0.32  (0.06 – 0.57) | 0.40  (0.14 – 0.66) | 0.26  (-0.01 – 0.52) | 0.50  (0.24 – 0.76) |
| phonemic verbal fluency | 0.16  (-0.10 – 0.42) | 0.61  (0.34 – 0.87) | 0.47  (0.21 – 0.74) | 0.54  (0.27 – 0.79) | 0.53  (0.26 – 0.79) |
| semantic verbal fluency | 0.37  (0.11 – 0.63) | 0.41  (0.41 – 0.67) | 0.35  (0.09 – 0.61) | 0.50  (0.24 – 0.76) | 0.33  (0.07 – 0.59) |

**Legend to supplementary table C**: MDD: Major Depressive Disorders; 95%-CI: 95% Confidence Interval
